# The negative effects of extracellular vesicles in the immune system

**DOI:** 10.3389/fimmu.2024.1410273

**Published:** 2024-09-20

**Authors:** Yang Wang, Cuifang Li, Feifeng Wu, Jueyi Mao, Junquan Zhu, Haotian Xie, Xin Zhou, Chuan Wen, Jidong Tian

**Affiliations:** ^1^ Department of Pediatrics, The Second Xiangya Hospital of Central South University, Changsha, China; ^2^ Department of Gastroenterology, The Second Xiangya Hospital of Central South University, Changsha, China

**Keywords:** extracellular vesicles, T cell, B cell, neutrophil, monocyte, macrophage, immune

## Abstract

Immunity is a critical self-defense mechanism of the human body, wherein immune cells and immune molecules play a crucial role. Extracellular vesicles (EVs), derived from immune cells or other cells, play a significant role in tumors, autoimmune diseases and other immune-related disorders by serving as carriers and facilitating intercellular communication through the transfer of cargoes. Numerous studies have revealed that EVs can exacerbate disease development by modulating immune responses. Therefore, this paper focuses on the effects of EVs on the number, activity and function of different types of immune cells and the release of immune molecules (such as cytokines, antigens, antibodies, etc) in various diseases, as well as the roles of EVs associated with different types of immune cells in various diseases. We aim to provide a comprehensive review of the negative effects that EVs play in the immune system to provide more ideas and strategies for the management of clinical immune diseases.

## 1 Introduction

Extracellular vesicles (EVs) represent a diverse class of vesicles that are present in various body fluids, including plasma, urine, saliva and milk. They can be produced by almost all eukaryotic cells. EVs encompass various subtypes of cell-released membrane structures such as exosomes, microvesicles, microparticles, ectosomes, oncosomes, apoptotic bodies and many other names (1). EVs protect their contents from degradation during recycling and are capable of transporting a variety of cargoes, including proteins, lipids, sugars and nucleic acids (2). EVs can directly bind to cellular receptors or transfer their contents to target cells, thereby triggering signaling and downstream transport, and regulating a range of pathophysiological functions in target cells, such as dynamic homeostasis, induction/inhibition of inflammation and promotion of repair (3).

The human body’s health is intimately linked with its immune system. The immune system is a complex network of immune organs, immune cells and immune molecules that work together to identify and remove foreign invading antigens and mutated or senescent cells in the body, thus maintaining a stable internal environment. Any abnormalities in the composition or function of the immune system can lead to immune diseases. In recent years, increasing evidence have highlighted the significant role of EVs in the pathogenesis of immune diseases (4).

EVs play a critical role in mediating communication between immune cells and other cells, as well as maintaining homeostasis in the body through multiple actions. Firstly, EVs can maintain homeostasis by regulating T cell (Treg) function. For instance, intrapericardial-injected exosomes were absorbed by major histocompatibility complex (MHC)-II+ antigen-presenting cells, and induced Foxo3 activation via modulating PP2A/p-Akt/Foxo3 pathway. Foxo3 promoted expression and secretion of Interleukin (IL)-10, IL-33, IL-34 to establish a Treg-inducing niche in the mediastinal lymph node. Following myocardial deployment, Treg orchestrate inflammation resolution and cardiac reparation (5). Additionally, small EVs containing surface-bound IL-2 have been shown to promote the recruitment of T cells, dendritic cells and macrophages in the tumor tissue, thereby enhanced the anticancer activity of T cells (6). Secondly, EVs can exert anti-inflammatory, anti-tumor and other effects by affecting macrophage polarization. For example, mesenchymal stem cells (MSCs)-derived EVs repolarized M1 pro-inflammatory macrophages to M2 anti-inflammatory macrophages by transmitting DUSP2 and DUSP3, effectively attenuated the inflammatory response in tendinopathy (7). Additionally, MSCs-EVs delivered miR-744-5p to block glioma progression by inhibiting macrophage TGFB1 expression and M2 polarization via miR-744-5p/TGFB1/MAPK axis (8). Thirdly, interactions between EVs and neutrophils have also been shown to maintain homeostasis. In asthmatic mouse model, micrococcus luteus-derived EVs significantly attenuated neutrophilic airway inflammation by decreasing IL-1β and IL-17 production in bronchoalveolar lavage fluid and the number of group 3 innate lymphoid cells in lung tissue (9). Additionally, after partial hepatectomy, liver-specific apoptotic EVs were released into the circulation and internalized by neutrophils to promote the secretion of hepatocyte growth factor, inducing a non-inflammatory, pro-regenerative neutrophil phenotype, and promoting liver regeneration (10). Circulating neutrophils released EVs containing mitochondria that carried superoxide dismutase 2, which reduced intravascular reactive oxygen species and maintained endothelial homeostasis, and acted as an anticoagulant in sepsis (11).

Recent studies have also shown that EVs can be used for vaccine development and drug delivery. Outer membrane vesicles (OMVs), which are EVs shed from bacterial outer membrane, have demonstrated a superior safety profile and the capacity to induce a full immune response, making them an attractive candidate for the development of mucosal vaccines (12). Furthermore, OMVs have also been considered as a promising drug delivery tool due to their inherent immune response properties, their accumulation in tumors and flexibility in engineering design (13).

Although EVs exhibit a non-negligible role in maintaining homeostasis and promoting repair in the organism, a large number of studies have indicated that EVs can also contribute to the development of diseases through their modulation of immunity. Therefore, this paper aims to provide a systematic review of the effects of EVs on various aspects of immune cells, including their number, activity, function and immune molecule release, as well as the role of immune cell-associated EVs in diseases to provide a more comprehensive review of the negative effects of EVs as in the immune system to provide more ideas and strategies for the management of clinical immune diseases.

## 2 Interaction between EVs and immune cells

### 2.1 EVs & T cells

T cell is one of the major cell types in the human immune system and primarily mediates cellular immunity. Additionally, EVs play an important role as immunomodulators in the communication between T cells and other cells.

On the one hand, EVs in the context of disease exacerbate disease pathology by attenuating T-cell responses. Firstly, tumor-derived EVs have been shown to evade immune surveillance by reducing the function or number of T cells (**Table 1**). For example, in gastric cancer cells, lysine-specific demethylase 1 inhibited T-cell activation and proliferation via exosomal programmed cell death 1 ligand 1, promoting the development of gastric cancer (14). Invasive breast cancer cell-derived EVs transferred transforming growth factor-β type II receptor to CD8+ T cells (15, 16), while chronic myeloid leukemia cell-derived EVs to T cells (17), promoting T cell depletion, resulting in the failure of immunotherapy. Additionally, myeloma cell line MOPC315.BM-derived EVs promoted myeloma cell escape and progression by modulating the lymphoid-like cell phenotype, in which CD4+ T cells exhibited pro-tumorigenic features, increasing the expression of immune checkpoint PD-1 and CTLA-4 while decreasing the expression of CD27, and facilitating the generation of an immunosuppressive environment (18). Interestingly, CD19+ leukemia cells NALM6-derived EVs carrying CD19 antigen could activate CD19 chimeric antigen receptor-T (CAR-T) cells, and transient activation dramatically promoted the release of a variety of inflammatory cytokines, including IL-2, IL-4, interferon (IFN)-γ, IL-10, IL-6, tumor necrosis factor (TNF)-α, IL2RA, IL-13, LTA and TNFSF18. However, sustained activation caused CD19 CAR-T cells to enter a depleted state, with increased inhibitory receptors PD1 and CTLA4, and significantly increased in the markers of exhaustion CD244 and CD160, and weakened their anti-tumor activity (19). Secondly, EVs are also believed to accelerate the progression of autoimmune diseases (**Table 1**). (1) In terms of the number of T cells, one research has demonstrated that lncRNA HOXA transcript at the distal tip was upregulated in rheumatoid arthritis (RA) synovial fibroblast cell-derived exosomes and promoted the expression of STAT3 by inhibiting the expression of miR-1908-5p, which increased the proportion of T-helper17(Th17) cells and decreased the proportion of Treg cells in the CD4+ T cell population, leading to increased synovial inflammation and cartilage erosion (20). Similarly, Schneider et al. found that in patients with juvenile idiopathic arthritis, the release of CD73-containing EVs from activated CD8+ T cells promoted adenosine production and inhibited T cell proliferation and function, which synergized with the high ATPase activity of the Treg cells and played a key role in terminating the immune response (21). (2) In terms of the function of T cells, Sun et al. reported that exosomes harvested from the sera of mice with experimental autoimmune myocarditis induced immunometabolic dysfunction of CD4+ T cells through cargo miR-142, thus promoted disease progression (22). These findings underscore the critical role of EVs in modulating T cell responses.

**Table 1:** Effect of EVs on immune cell composition and function in immune diseases.

Disease	Source	Cargo	Targeted cell	Direct effect	Final effect	Ref.
Lumpy disease	Alveolar lavage fluid	–	Monocytes	IL-1β,IL-6,TNF, CCL2 and Reactive Oxygen↑	converge monocytes and T cells	(35)
SARS-CoV-2 pneumonia	SARS-CoV-2 Spike transfected cells	miR-148a	Microglial cells	USP33↓, IRF9↓	Severe neuroinflammation within the central nervous system	(36)
Preeclampsia	Trophoblast cells	–	Macrophages	TNF-α and IL-6↑, promote M1 polarization	Produce high blood pressure and proteinuria	(38)
Crohn’s disease	Intestinal epithelial cells	–	Macrophages	Pro-inflammatory cytokine secretion↑	Trigger pro-inflammatory response and amplify replication	(40)
Crohn’s disease	Intestinal epithelial cells	mtDNA and nDNA	Macrophages	STING pathway↑	Promote intestinal inflammation	(39)
Diabetic nephropathy	Lipotoxic renal tubular epithelial cells	LRG1	Macrophages	TGFβR1↑, activate macrophages	Promote renal inflammation and apoptosis	(41)
Obesity	Adipocytes	miR-34a	Macrophages	KLF4↓, inhibited M2 polarization	Exacerbate obesity-related systemic inflammation and metabolic dysregulation	(42)
Rheumatoid arthritis	Mouse synovial fibroblasts	HOTTIP	T cells	Th cells↑, Treg cells↓	Exacerbate synovial inflammation and cartilage erosion	(20)
	Patient’s plasma	–	B cells	Activation marker expression↓, calcium mobilization↓, tyrosine phosphorylation↓		(26)
Myocarditis	Mouse serum	miR-142	CD4+T cells	MBD2 and SOCS1↓	Affect glycolytic pathways and promote inflammatory responses	(22)
Gastric cancer	Gastric cancer cells	PD-L1	T cells	PD-L1 expression↑, T cell response↓	Promote tumor development	(14)
		miR-519a-3p	Macrophages	MAPK/ERK pathway↑, M2 polarization↑	Promote angiogenesis and liver metastasis	(48)
Invasive breast cancer	Invasive breast cancer cells	TβRII	CD8+T cells	TGF-β/SMAD3 pathway↑	CD8+ T cell depletion and anti-tumor immune dysregulation	(15, 16)
Chronic myeloid leukemia	Chronic myeloid leukemia cells	–	T cells	T cell activation↓, cytokine secretion↓	T cell metabolism tends to be quiescent	(17)
CD19+ tumor	CD19+ tumor cells	CD19+ antigen	CD19 CAR-T cells	Cytokine release syndrome	CD19 CAR-T cell depletion and diminish anti-tumor activity	(19)
Nasopharyngeal cancer	Nasopharyngeal cancer cells	RNF126	Macrophages	PTEN↓, PI3K/AKT pathway↑, M2 polarization↑	Promote tumor growth and metastasis	(43)
Glioma	Glioma cells	miR-3184-3p	Macrophages	Target PDCD4, induce M2 polarization	Aggravate tumor malignancy	(44)
		miR-10b-5p	Macrophages	Target the NEDD4L/PIK3CA/AKT axis, promote M2 polarization	Promote tumor proliferation, migration, and invasion	(45)
Glioblastoma after radiotherapy	Glioblastoma cells after radiotherapy	circ-0012381	Microglial cells	miR-340-5p↓, M2 polarization↑	Reduce phagocytosis and promote tumor proliferation	(46)
Pancreatic cancer	Pancreatic cancer cells	FGD5-AS1	Tumor-associated macrophages	STAT3/NF-κB pathway↑, M2 polarization↑	Promote proliferation, migration and invasion	(47)
Melanoma	Melanoma cells	rHSP90α	Monocytes	HLADR expression↓	Enhance anti-apoptotic capacity	(49)

EVs, Extracellular vesicles; IL, Interleukin; TNF, Tumor necrosis factor; miR, microRNA; USP33, Ubiquitin specific peptidase 33; IRF9, Interferon regulatory factor 9; LRG1, Leucine-rich α-2-glycoprotein 1; TGFβR1, Transforming growth factor-β type 1 receptor; HOTTIP, HOXA transcript at the distal tip; Th, T-helper; Treg, Regulatory T; PD-L1, Programmed cell death 1 ligand 1; TβRII, Transforming growth factor-β type II receptor; CAR-T, Chimeric antigen receptor-T; NF-κB, Nuclear factor-κB; rHSP90α, Recombinant heat-shock protein 90. Symbol “↑” means that the expression or secretion of certain factors or pathways are increased. Symbol “↓” means that the expression or secretion of certain factors or pathways are reduced.

On the other hand, T cells are not only the target cells of EVs, but also can secrete EVs into the target cells to alter or impair biological functions of target cells, leading to disease development. First of all, T cell-derived EVs can induce disease onset and progression by impairing the function of epithelial or endothelial cells. The Ca2+ and cAMP pathways are the major signaling systems of the secretory epithelium and control almost all secretory gland functions, and Ca2+ signaling is essential for controlling fluid and enzyme secretion from exocrine glands. It has been indicated that miR-142-3p was highly expressed in salivary glands of Sjögren’s syndrome patients, including inflammation-infiltrated T cells, which secreted exosomes containing miR-142-3p that significantly down-regulated key components of Ca2+ signaling and cAMP production in salivary gland epithelial cells—SERCA2B, RyR2 and AC9—limited cAMP production, altered calcium signaling, reduced protein production in salivary gland cells, ultimately directly impaired epithelial cell function (23). Furthermore, CD4+ T lymphocytes-derived exosomes transported MEK1/2 and ERK1/2, and activated NADPH oxidase in cardiac microvascular endothelial cells, which played a key role in the pathogenesis of angiosclerosis and coronary microcirculatory dysfunction (24). Secondly, T cell-derived EVs could also cause the disorder of the endocrine system. For instance, exosomes released from T cells contained specific microRNAs that triggered chemokine expression and apoptosis in recipient pancreatic β cells, increasing the incidence of diabetes in non-obese diabetic mice (25).

In conclusion, these studies suggested that T cells can play an immunomodulatory role through EVs regardless of whether they are the source or target cells of EVs; however, T cell subtypes are varied. The specific regulatory mechanisms between EVs and T cell subtypes in different disease settings still need more scientific exploration.

### 2.2 EVs & B cells

B cells stimulated by antigen can differentiate into effector B cells, which subsequently produce antibodies to mediate humoral immunity. In addition, B cells participate in immune regulation via presenting antigens and secreting cytokines.

It has been observed that when the body is in a pathological condition, EVs can release their contents into B cells, which affects the function of B cells. For example, RA is a chronic autoimmune inflammatory disease. B cells are the central component of the immune pathology of RA. Rincón-Arévalo et al. investigated the effect of medium/large EVs (m/lEVs) and m/lEVs-forming immune complexes from RA patients on B cell activation. They found that these EVs reduced the expression of activation markers, diminished calcium mobilization and decreased tyrosine phosphorylation in activated B cells *in vitro* (**Table 1**) (26). These findings suggested that EVs may contribute to the dysregulation of B cell function in RA.

In addition, mixed cryoglobulinemia is a systemic vasculitis mediated mainly by the immune complex and is the most common extrahepatic manifestation associated with hepatitis C virus (HCV), characterized by B cell proliferation and autoantibody production, in which HCV-associated exosomes have been shown to induce B cell activating factor upregulation in macrophages through activation of Toll-like receptor 7 and Nuclear factor-κB (NF-κB), accompanied by phosphorylation of the p65 subunit, stimulating B cell activation to produce excessive anti-HCV antibodies and then leading to the development of mixed cryoglobulinemia symptoms (27).

The above studies suggested that there may be a dual role for EVs in B cell activation, which meant that the effect of EVs on B cell immunoregulatory function in specific diseases remains largely unknown.

### 2.3 EVs & neutrophils

Neutrophils are the most abundant granulocyte type, and they are the main effect cells of the body against infection by extracellular pathogens and can be recruited from the blood to act at the site of inflammation of infection. In addition, interactions between EVs and neutrophils have been shown to influence immune regulation.

Lung EVs have been shown to contain protein and dsDNA components, both of which played a critical role in the recruitment of neutrophils during the early stages of bacterial infection (28). In another study, pyroptotic bodies (micron-sized EVs) were characterized in the alveolar lavage fluid during the early phases of acute lung injury. This study confirmed that the release of caspase-1-mediated pyroptosis-dependent vesicles from alveolar macrophages in the early stages of lipopolysaccharide-induced acute lung injury in mice. These EVs encapsulated several injury-associated molecular patterns—including TNF-α, IL-6, IL-1β, fibronectin, HMGB1, heat shock proteins, and s100s—promoting the activation of lung epithelial cells through the p38 MAPK signaling pathway, which led to interstitial vascular edema, recruited neutrophils and participated in uncontrolled inflammatory responses (29).

Neutrophil-derived EVs can cause tissue cell dysfunction and inflammatory chemotaxis, thereby contributing to the development of autoimmune diseases. Firstly, in terms of endothelial cell injury, activated neutrophil-derived EVs containing miR-142-3p and miR-451 have been found to target endothelial cells to trigger an inflammatory cascade reactions and induce direct vascular injury in anti-neutrophil cytoplasmic antibody-associated small vessel vasculitis (30). Moreover, the exosomes in microscopic polyangiitis have also been shown to induce endothelial damage (31). Secondly, in terms of inflammatory chemotaxis, a study by Shao et al. revealed that neutrophils from patients with generalized pustular psoriasis secreted more exosomes than those from healthy individuals, which were then rapidly internalized by keratinocytes. This resulted in the increased expression of inflammatory molecules through the activation of NF-κB and MAPK signaling pathways, exacerbating neutrophil migration and the formation of an autoinflammatory cycle in generalized pustular psoriasis (32).

Generally speaking, EVs can indeed promote the recruitment and migration of neutrophils, but to some extent, it can lead to self inflammatory cycle and cause inflammatory cascade reaction. Further research is needed to clarify the exact mechanism of neutrophil immunoregulation of EVs in diseases.

### 2.4 EVs & monocytes/macrophages

Monocytes originate from the bone marrow, circulate to the whole body through the blood, and differentiate into macrophages, which play an essential role in immune surveillance and response (33). In addition to direct cell-cell communication, monocytes and macrophages also communicate with other cells through EVs for intercellular communication. The communication mediated by these EVs can also impair immune function and contribute to the pathogenesis of various diseases (33, 34).

**Figure 1:**
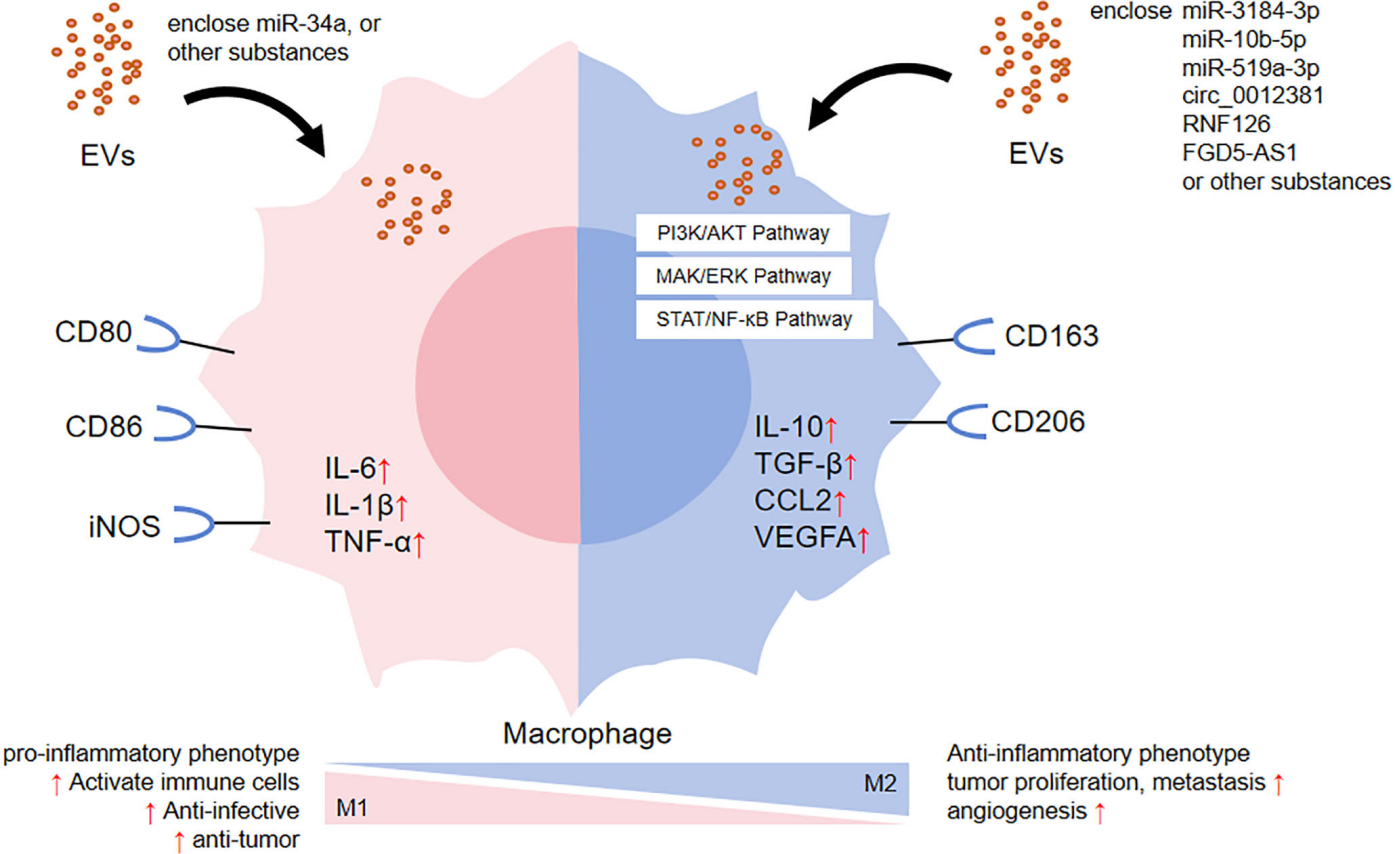
The effect of EVs on macrophage polarization.

On the one hand, EVs have been found to modulate the phenotype and function of monocytes or macrophages, which has a significant impact on the immune function of the body (shown in **Figure 1**). First of all, EVs can induce the immune state of the body toward a pro-inflammatory direction, as demonstrated in **Table 1**. (1) In the respiratory system, It has been shown that exosomes presented in alveolar lavage fluid from patients with Lumpy disease induced elevation of IL-1β, IL-6, TNF, CCL2 and reactive oxygen species in monocytes, demonstrating the effect of exosomes on nodular inflammation (35). In addition, SARS-CoV-2 Spike transfected cells could activate microglia through exosomal miR-148a to target the Ubiquitin Specific Peptidase 33/Interferon regulatory factor 9 axis, leading to severe neuroinflammation in the central nervous system (36). (2) In the nervous system, in patients with multiple sclerosis, neurons secreted EVs containing interferon stimulated gene 15 protein. Interferon stimulated gene 15 protein directly activated microglia in a CD11b-dependent manner and promoted the production of chemokines, inflammatory factors and iNOS in microglia (37). (3) In the circulatory system, EVs secreted by trophoblast cells in the placenta of patients with preeclampsia can act as key inflammatory signals driving macrophage M1 polarization to a pro-inflammatory phenotype. It has been shown that trophoblast-derived EVs in preeclampsia women significantly up-regulated macrophage M1 gene markers, such as IL-1β, IL-6 and TNF-α, and significantly down-regulated macrophage CD163 expression compared to trophoblast-derived EVs in normal pregnant women. These results suggested that trophoblast-derived EVs in preeclampsia may be a key inflammatory signal driving macrophage polarization toward pro-inflammatory phenotypes (38). (4) In the digestive system, Crohn’s disease-related EVs were transported from intestinal epithelial cells to macrophages, which not only triggered the inflammatory response (39), but also expanded the replication of secondary bacterial infections in host cells (40). (5) In the urinary and endocrine systems, Leucine-rich α-2-glycoprotein 1-rich lipotoxic renal tubular epithelium-derived EVs induced inflammation and injury in diabetic nephropathy by activating macrophages through a TGFβR1-dependent process (41). Additionally, adipocytes delivered miR-34a to neighboring macrophages via exosomes, resulting in a shift to a pro-inflammatory M1 phenotype that exacerbated obesity-associated systemic inflammation and metabolic dysregulation (42).

Secondly, EVs have been shown to play a role in promoting tumor progression through various oncogenic mechanisms (**Table 1**). (1) Immunosuppression. For instance, the nasopharyngeal carcinoma cell-derived exosomes RNF126 (43), glioma cell-derived EVs miR-3184-3p (44) and miR-10b-5p (45) altered the tumor immune microenvironment and aggravated tumor progression by up-regulating the expression of M2 polarization-associated factors IL-10, TGF-β, CD163, and Arg-1, as well as down-regulated the expression of M1 polarization-associated factor TNF-α. (2) Proliferation. For example, after radiotherapy, glioblastoma cell exosomal circ_0012381 could induce microglia M2 polarization, reduce their phagocytosis and then promote tumor proliferative capacity (46). Pancreatic cancer cell exosomal FGD5-AS1 have been shown to induce M2 polarization of tumor-associated macrophages, resulting in increased proliferation, migration and invasion (47). (3) Angiogenesis. As shown by gastric cancer cell exosomal miR-519a-3p, which mediated angiogenesis by inducing M2 polarization in intrahepatic macrophages, thus promoted liver metastasis (48). (4) Anti-apoptosis. For example, melanoma cell EVs recombinant heat shock protein 90α could enhance the resistance of monocytes to apoptosis (49).

On the other hand, EVs released by monocytes or macrophages can also aggravate the severity of diseases through different mechanisms. Firstly, they can induce tissue cells damage. For example, in the exosomes secreted by macrophages treated with HIV virus protein, miR-23a and miR-27a impaired the tight junction barrier and mitochondrial function of lung epithelial cells respectively, increasing the susceptibility to alveolar infection and injury (50). Macrophage-derived EVs containing high levels of leucine-rich α-2-glycoprotein 1 (51) or tumor necrosis factor-related apoptosis-inducing ligand (41) have been shown to induce renal tubular epithelial cell injury and apoptosis via corresponding transforming growth factor β receptor 1 (51) or death receptor 5 (41) dependent processes. Secondly, EVs can enhance tumor cell resistance. For instance, the tumor-associated macrophage-derived exosomal lncMMPA promoted tumor proliferation by activating the glycolytic pathway in hepatocellular carcinoma cells (52). MiR-222-3p, an M2 macrophage-derived EVs cargo, mediated gemcitabine resistance in pancreatic cancer (53). Thirdly, EVs can also modulate other immune cells. In the animal model of Guillain-Barré syndrome, M1 macrophage-derived exosomes exacerbated the disease by directly regulating the differentiation (increased proportion) and function (increased IFN-γ intensity) of Th1 effector cells (54). Finally, EVs can inhibit precursor cell differentiation. For example, miR-98, an M1 macrophage-derived exosomal cargo, was transferred to MC3T3-E1 cells and exacerbated bone loss in osteoporosis by inhibiting osteogenic differentiation through downregulation of DUSP1 and activation of the JNK signaling pathway (55).

Although a large number of studies have shown that EVs promoted diseases progression by inducing M2 polarization of macrophages, M2 macrophages could be subdivided into four subtypes, so more future researches are needed to clarify the more accurate influence of EVs on M2 macrophage subtypes.

## 3 Association among EVs, immune molecules and immune diseases

Immune molecules, such as cytokines, antibodies, antigens, complements, MHC and other substances, are synthesized and secreted by immune cells to regulate the immune response. Many evidences have suggested that EVs can play an immunomodulatory role by promoting the secretion or delivery of immune molecules.

Specific proteins in plasma (56) and tear-derived (57) exosomes from patients with autoimmune thyroid disease promoted the secretion of TNF-α (56) and inflammatory cytokines (IL-6, IL-8 and MCP-1) (57) respectively, activating inflammatory responses, orbital tissue remodeling and fibrosis. Plasma-derived EVs from patients with dermatomyositis triggered the release of STING phosphorylated pro-inflammatory cytokines, including IFN-β, TNF-α and IL-6, and the severity of the disease correlated with the levels of these cytokines (58). In addition, after immunization of mice with exosomes from the cerebrospinal fluid of patients with antibody-positive autoimmune encephalitis, Gu et al. showed an increase in the frequency of neuronal antigen-specific inflammatory cytokines IFN-γ and IL-17 production (59). Similarly, it has been shown that patients with minimal hepatic encephalopathy (60) and preeclampsia (61) have a pro-inflammatory state, and their circulating EVs altered the body toward a pro-inflammatory state by up-regulating the expression of pro-inflammatory cytokines IL-17, IL-21 and others.

In a study of antibody-mediated allograft rejection, it was found that plasma-derived EVs led to the upregulation of C3 gene expression and downregulation of CFH expression in renal tubular epithelial cells, and deposition of C4d in endothelial cells, these had pro-inflammatory, pro-aging, and pro-fibrotic effects (62). Another study *in vivo* showed that mouse skin and heart allografts as well as human skin grafts transferred cell-free donor MHC antigens to secondary lymphoid tissues via EVs, triggering upregulation of calcium fluxes and CD86 in alloreactive B cells and eliciting B-cell alloimmunity (63). Similarly, using a mouse heart transplantation model, Liu et al. found that donor dendritic cells transferred EVs with functional MHC antigens to recipient lymphoid tissues, and recipient dendritic cells were activated by acquisition of EVs and directly presented to allogeneic reactive T cells (64).These suggested that EVs may play a role in mediating allograft rejection, serve as noninvasive diagnostic biomarkers of allograft rejection, and promise potential therapeutic targets for the prevention of allograft rejection. Heart transplant patients were divided into two groups according to whether they developed rejection within 1 year: the group that developed rejection and the group that did not develop rejection. The surface protein profiles of plasma-derived EVs of the two groups were studied, which revealed that the acute cellular rejection response differentiation factors CD3, CD2, ROR1, SSEA-4, HLA-I and CD41b; and the antibody-mediated rejection differentiation factors HLA-II, CD326, CD19, CD25, CD20 (65).In renal transplantation, detection of T-cell-derived EVs in urine samples from renal transplant patients by the iKEA method demonstrated the highest differential expression of CD3 in transplant-rejected patients compared to patients with no signs of rejection on renal biopsy (66). Regarding antibody-mediated rejection, the gene combination scores of the four genes gp130, SH2D1B, TNF-α, and CCL4 were significantly higher in plasma exosomes of HLA-sensitized renal transplant recipients than in cellular rejection responders and non-rejection responders. Thus, mRNA transcriptional profiles based on the four genes mentioned above in plasma exosomes can be used to predict ongoing and/or impending antibody-mediated rejection reactions (67).

In addition, it has been found that the cerebrospinal fluid EVs of autoimmune encephalitis and herpes simplex encephalitis had different properties. Herpes simplex virus-induced exosomes played a key role in the development of herpes simplex encephalitis by delivering surface/cellular neuronal autoantigens to trigger brain autoimmunity (68).

## 4 Discussion

The immune system is an interactive network of immune organs, immune cells, and immune molecules (69). The immune system disorders can occur if there is a defective or overactive immune function in the body. As important mediators of intercellular communication, the role of EVs in immune regulation has attracted much attention in recent years. Although EVs play a variety of positive effects in immune responses, the negative effects cannot be ignored as well. As mentioned earlier, several studies have demonstrated the involvement of EVs in the development of immune diseases, including tumors (14–19), autoimmune diseases (20–23), allograft rejection (62–67), and so on. Not only can non-immune cell derived EVs aggravate diseases by affecting the number and function of immune cells and the release of immune molecules, but immune cell derived EVs can also participate in the occurrence and development of diseases by suppressing immunity, promoting tissue and cell damage, and triggering excessive immune response.

Although some studies have revealed the negative role of EVs in the immune system, there is still some controversy in this area. The immunomodulatory role of EVs is very complex and has not been fully elucidated. Different sources and compositions of EVs may have different effects in the same immune environment. The function of EVs can be influenced by a variety of factors, such as source cell type, composition, the microenvironment in which they are found, time factor, the method of isolation and purification, etc. Traditional batch analysis methods often fail to dissect the inherent heterogeneity in EVs population, so various methods for single extracellular vesicle analysis have been introduced in recent years, such as nanoflow cytometry, the ExoView platform, super-resolution fluorescence imaging, single-particle dark-field imaging, single particle automated Raman trapping analysis, and so on (70). The complexity of EVs makes the reproducibility and consistency of research results a great challenge. In addition, most of the current researches on the negative effects of EVs were based on *in vitro* experiments or animal models. There is a lack of direct evidence for clinical applications, so how to translate laboratory research into clinical application still needs to be further explored.

Therefore, the future direction can be developed in several directions. Firstly, the development of more advanced isolation and analytical techniques to improve the purity and identification accuracy of EVs and ensure the reliability and repeatability of research results will also be an important direction for future research. Secondly, the specific action mechanism of EVs in immune regulation should be further explored, including their interactions with immune cell subtypes, signaling pathways, and functional changes at different stages of different disease states. In addition, large scale clinical studies should be conducted to verify the effectiveness and safety of EVs as biomarkers and therapeutic targets.

## 5 Conclusion

In conclusion, despite numerous studies exploring the mechanisms of EVs in immune diseases, their scope remains limited, and there are several disease areas and developmental mechanisms that require further in-depth and detailed investigation.
